# A Severe Case of *Arthrographis kalrae* Keratomycosis

**DOI:** 10.1155/2013/851875

**Published:** 2013-11-27

**Authors:** Siti Roszilawati Ramli, Alex Lourdes Francis, Yushaniza Yusof, Tzar Mohd Nizam Khaithir

**Affiliations:** ^1^Institute for Medical Research, Jalan Pahang, 50588 Kuala Lumpur, Wilayah Persekutuan, Malaysia; ^2^Pathology Department, Hospital Raja Permaisuri Bainun, Jalan Hospital, 30990 Ipoh, Perak, Malaysia; ^3^Ophthalmology Department, Hospital Seri Manjung, 32040 Manjung, Perak, Malaysia; ^4^Department of Medical Microbiology and Immunology, Universiti Kebangsaan Malaysia Medical Centre, 56000 Kuala Lumpur, Wilayah Persekutuan, Malaysia

## Abstract

A 52-year-old man with diabetes developed a unilateral central corneal ulcer after accidental foreign body inoculation. He complained of pain and loss of visual acuity in the injured eye, which displayed redness and edema and eventually discharged pus. A corneal scraping from the left eye orbit revealed fungal elements, and cultures of the material grew a fungus. The isolate was identified as *Arthrographis kalrae* based on gross and microscopic morphologies. The patient received amphotericin B intravenously and itraconazole orally. The wound healed following surgical intervention, but the patient lost the use of his left eye.

## 1. Case Report

A 52-year-old gentleman and a known case of diabetes mellitus presented with left eye pain for 1-week duration after accidental foreign body inoculation while riding his motorcycle. The eye was red, watery, and vision was reduced 3 days before admission. He was referred to Hospital Seri Manjung after seven days, when treatment with chloramphenicol ointment by a nearby government clinic failed.

His left vision was compromised, 6/12 on glasses and 6/18 unaided compared to 6/9 with glasses and 6/6 unaided in his right eye. Slit lamp examination of the left cornea disclosed corneal ulcer with epithelial and stromal infiltrates and surrounding edema along with endothelial plaque. Epithelial abrasion was also present measuring 4 mm long and 3.8 mm wide. The corneal sensation in the left eye was reduced. There was also meibomitis in both eyes. A diagnosis of infectious keratitis was made. Both systemic and topical antifungal and antibiotics were prescribed; that is, tablet vibramycin 100 mg daily and tablet fluconazole 200 mg daily together with hourly intensive gentamycin, cefuroxime, amphotericin B, fluconazole, and acyclovir eye drops 5 times a day were started. After a week, the left eye stromal infiltrates and the endothelial plaques showed gradual reduction with treatment. The patient asked for a discharge against medical advice, and after a few days, he came back as there was rapid deterioration with formation of dense stromal abscess, hypopyon and haziness at the superior aspect of the cornea and secondary glaucoma. The corneal epithelial defect had increased in size to 8 mm wide and 9.6 mm long.

**Figure 1:**
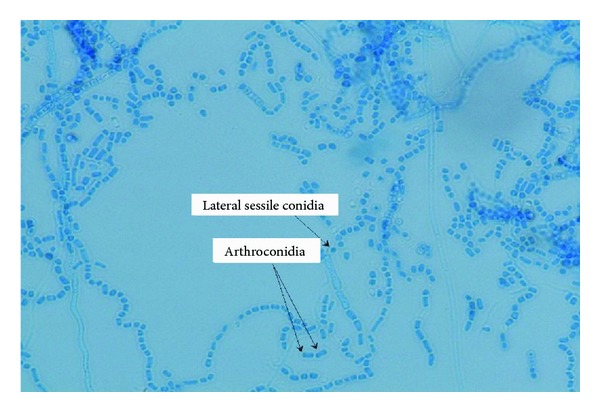
Microscopic examination of corneal scrape material stained with lactophenol cotton blue, demonstrating the presence of arthroconidia and lateral sessile conidia of hyaline fungal hyphae (×100 magnification).

**Figure 2:**
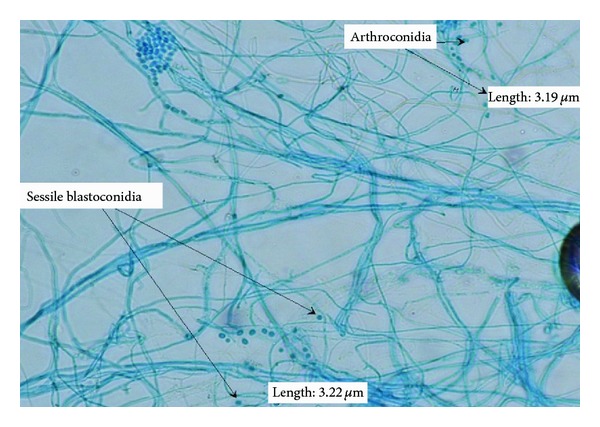
Microscopic examination of corneal scrape material stained with lactophenol cotton blue, demonstrating the presence of arthroconidia and spherical blastoconidia (×100 magnification).

Corneal scraping showed presence of few pus cells; however, no organism was noted. Culture grew a fast growing mould.Colonies on Sabouraud dextrose agar (SDA) at 30°C were cream coloured and appeared pasty. However, after 24 hours, they became dry and velvety, and after a further 48-hour incubation, the colonies became cottony with a distinct yellow on the reverse of the plate. Similar morphology was seen on potato dextrose agar (PDA). On microscopy, the yeast phase was not well defined as stated in the literature. The filamentous phase showed hyaline, septate hyphae, dendritic (tree-like) conidiophores, and chains of rectangular arthroconidia 2–4 *μ*m in length not separated by disjunctor (intervening) cells (Figure 1). The vegetative hyphae also produced sessile, subglobose conidia measuring 4 × 5 *μ*m (Figure 2). Based on the detailed study of the macroscopic and microscopic morphological characteristics of the cultures, the isolates were identified as *Arthrographis kalrae*.

Despite the intensive treatment that the patient received, his condition rapidly worsened where his hypopyon increased to almost fill almost the total anterior chamber of the eye within 48 hours. The cornea eventually perforated, and he required a full thickness cornea graft to control the infection. Unfortunately, despite the continuation of the intensive systemic and topical treatment postoperatively, the cornea graft failed. He is still under our followup with the vision of only perception to light.

## 2. Discussion

Mycotic keratitis or keratomycosis is a fungal infection of the cornea. It most commonly occurs in male, with history of wearing contact lenses and/or outdoor trauma involving the eye. Other causes of mycotic infections of the eye may be climate, preexisting diseases (either ophthalmic or systemic), and inappropriate use of topical or systemic antibiotics and steroids [1]. *Arthrographis kalrae* is an uncommon etiology in fungal infections [2].

*Arthrographis kalrae* previously named *Oidiodendron kalrai *in 1976 has been reported an ocular pathogen causing severe keratomycosis; however, the occurrence is very rare [2]. *Arthrographis kalrae *can commonly be isolated from soil and compost. There are a few publications on infection of *A. kalrae *on human, which included reports on refractory arthritis, onychomycosis, mycetoma, meningitis in immunocompromised, sinusitis and ophthalmitis, vasculitis, endocarditis and lung infections. There are three reports on *A. kalrae *keratitis, and all cases showed that it is frequently implicated by ocular trauma by foreign body inoculation besides all cases are associated with contact lenses. The clinical presentations were similar to *Acanthamoeba* keratitis with history of photophobia [3–12].

Our patient was a diabetic middle-age man who presented after a week of treatment failure and denied of photophobia. There was history of ocular trauma by foreign body inoculation; however, he was not wearing any contact lenses. His ocular presentation was mixture of feathery grey-white stromal infiltrates and epithelial plaque which resembled the presentation of bacterial suppuration involving the entire cornea. Thus, the clinical diagnosis of mycotic keratitis was superficial and difficult initially [13].

The diagnosis was made after the identification of *A. kalrae *by colony and microscopic morphologies of the cornea scrapping cultures. Although the identification of *A. kalrae* on conventional tests required experienced and knowledge on morphological characteristics, confirmation by polymerase chain reaction and sequencing may be used when conventional tests do not yield positive results [13].

Most *A. kalrae* keratitis reports showed successful treatment with amphotericin B, itraconazole, ketoconazole and voriconazole; our patient however only undergone suboptimal medical treatment which showed positive responds initially [10–12]. His condition however deteriorated since he discontinued treatment, besides poor host defense mechanism due to underlying uncontrolled diabetic mellitus.

## 3. Conclusion

*Arthrographis kalrae* has been reported an ocular pathogen causing severe keratomycosis, and here, we report a case of fungal keratitis, following foreign body inoculation in an uncontrolled diabetic patient. To our knowledge, this is the first report in Malaysia and among the limited number of reports worldwide. Although the occurrence is rare, the diagnosis should be suspected in cases with history of foreign body or soil inoculation to the eye and not responsive to antibiotics. Identification of *A. kalrae* can be difficult; hence, knowledge on morphological characteristic is deemed important, besides further confirmation by molecular investigations.

## References

[1] Foster CS (1992). Fungal keratitis. *Infectious Disease Clinics of North America*.

[2] Tewari RP, Macpherson CR (1971). A new dimorphic fungus, *Oidiodendron kalrai*: morphological and biochemical characteristics. *Mycologia*.

[3] Boan P, Arthur I, Golledge C, Ellis D (2012). Refractory *Arthrographis kalrae* native knee joint infection. *Medical Mycology Case Reports*.

[4] Xi L, Fukushima K, Lu C, Takizawa K, Liao R, Nishimura K (2004). First case of *Arthrographis kalrae* Ethmoid sinusitis and ophthalmitis in the People’s Republic of China. *Journal of Clinical Microbiology*.

[5] Chin-Hong PV, Sutton DA, Roemer M, Jacobson MA, Aberg JA (2001). Invasive fungal sinusitis and meningitis due to *Arthrographis kalrae* in a patient with AIDS. *Journal of Clinical Microbiology*.

[6] de Diego Candela J, Forteza A, García D (2010). Endocarditis Caused by *Arthrographis kalrae*. *Annals of Thoracic Surgery*.

[7] Degavre B, Joujoux JM, Dandurand M, Guillot B (1997). First report of mycetoma caused by *Arthrographis kalrae*: successful treatment with itraconazole. *Journal of the American Academy of Dermatology*.

[8] Sugiura Y, Hironaga M (2010). Arthrographis kalrae, a rare causal agent of onychomycosis, and its occurrence in natural and commercially available soils. *Medical Mycology*.

[9] Vos CG, Murk J-LAN, Hartemink KJ, Daniels JMA, Paul MA, Debets-Ossenkopp YJ (2012). A rare pulmonary infection caused by *Arthrographis kalrae*. *Journal of Medical Microbiology*.

[10] Perlman EM, Binns L (1997). Intense photophobia caused by *Arthrographis kalrae* in a contact lens- wearing patient. *American Journal of Ophthalmology*.

[11] Biser SA, Perry HD, Donnenfeld ED, Dosbi SJ, Chaturvedi V (2004). *Arthrographis* keratitis mimicking acanthamoeba keratitis. *Cornea*.

[12] Thomas BC, Zimmermann S, Völcker H-E, Auffarth GU, Dithmar S (2011). Severe *Arthrographis kalrae* keratomycosis in an immunocompetent patient. *Cornea*.

[13] Thomas PA, Kaliamurthy J (2013). Mycotic keratitis: epidemiology, diagnosis and management. *Clinical Microbiology and Infection*.

